# Cryogel Addition Effect on Ultrasound-Assisted Thawing of Pork Meat

**DOI:** 10.1155/ijfo/9662782

**Published:** 2024-12-17

**Authors:** Jonathan Coria-Hernández, Rosalía Meléndez-Pérez

**Affiliations:** Laboratory 13 Thermal and Structural Analysis of Materials and Foods, Multidisciplinary Research Unit, National Autonomous University of Mexico-Superior Studies Faculty at Cuautitlan (UNAM–FESC), Cuautitlan Izcalli, Mexico State, Mexico

## Abstract

The use of new technologies that allow for improving conventional food preservation processes is what the industry has been adopting in recent decades, with high-intensity ultrasound (US) and the application of cryoprotectant agents (cryogels) being those that have become more relevant today. For this reason, in this study, cuts of *Longissimus thoracis* pork frozen in liquid nitrogen with and without waxy starch cryogel and thawed under controlled conditions in water immersion and with US were used, evaluating thermal parameters such as the initial zone and the melting rate of ice crystals and quality parameters such as pH, water holding capacity (WHC), microstructure, color profile, shear force, and surface changes. It was shown that the addition of cryogel modifies the initial fusion zone, that US-assisted thawing increases the fusion rate, and that both factors influence the quality parameters. However, the main effect on pH is the use of cryogel, unlike WHC, color parameters, and shear force, where the main impact is the thawing method. These results conclude that waxy starch cryogel and the US at 50% thawing have the potential to apply assistance technology in food processing.

## 1. Introduction

Most meat products are produced, stored, and transported frozen. However, its processing or consumption requires the thawing process. The rate of which impacts the quality of thawed meat [1]. Thawing can be described as a process where frozen food is produced to a temperature without residual ice [2]. This process is mainly governed by heat transfer that promotes the existence of physical, chemical, and or biological changes, which involve alterations in its appearance, texture, chemical composition, taste, and nutritional value. When frozen foods are thawed using air or water, the surface of the ice melts to form a layer of water. Theoretically, water has lower thermal conductivity and diffusivity than ice. Therefore, this surface layer of water significantly reduces the rate at which heat is applied to frozen foods. In this sense, an insulating effect is promoted, which increases as the water layer of the thawed food becomes more abundant. On the contrary, during the freezing process, the increase in the thickness of the ice causes the heat transfer to accelerate due to the higher values in the thermal conductivity of the ice. Therefore, thawing is a substantially more prolonged process than freezing when differences in temperature and other conditions are similar [3, 4]. Most thaw systems deliver heat to the surface and then rely on conduction to transfer that heat to the center of the meat. When selecting a thawing system, a balance must be related between processing time, food quality, and processing problems such as effluent disposal, as gained during freezing may be compromised during thawing. Of these factors, the thaw time is the main criterion governing the selection of the system. This time depends on factors related to the product and environmental conditions. It includes the dimensions and shape of the product, particularly thickness, change in enthalpy, the thermal conductivity of the product, initial and final temperatures, surface heat transfer coefficient, and temperature of the thawing medium [5, 6]. There are two basic thaw methods, and it is crucial to be clear that the selection of equipment and process to use must consider the costs and limitations of each. (a) Thermal: These include equipment by hot air flow, immersion in water, or surface treatment with steam [7]. They depend on conventional heat conduction through the surface. (b) Electrical: They include microwaves, radiation, electric pulses, and ultrasound (US), which employ heat generation within the product. In this sense, authors such as Hu, Zhang, and Mujumdar [8]; Hu et al. [9]; and Cao et al. [10] have worked on the effect of infrared and microwave alternative thawing on frozen pork, fish, and yak meat; Bedane et al. [11] in radiofrequency and electric field thawing in chicken and squid; and Jia et al. [12], Xu et al. [13], and Guo et al. [14] with the evaluation of changes of US-assisted thawing in lamb meat quality.

The mechanisms of action of the US are classified as thermal (generation of heat or mechanical energy) and nonthermal (cavitation, rarefaction, formation of free radicals, micromechanical shocks, and radiation force). Cavitation is induced at 20–100 kHz in a liquid medium and is considered the basic principle of action. The cavitation bubbles generate rapid destruction and release high energies, leading to physical and chemical changes in biological structures. When the US propagates in a medium, the intensity or amplitude of the wave decreases with distance due to attenuation. Attenuation results from the absorption of energy by the medium. The energy loss is due to reflection, refraction, diffraction, and dispersion phenomena. Energy absorption will depend on the nature of the medium and can provide information about its physical properties. In gases and liquids, the most important mechanisms that contribute to the absorption of ultrasonic energy are viscosity and the phenomena of thermal conduction and thermal and structural relaxation, which are transformed into kinetic energy and internal energy of the molecules. In solids, the explanation is more complex due to several types of interactions. Reflection and refraction usually occur at the boundaries between zones of different impedance. Diffraction occurs due to the presence of interposed barriers in the wave direction, while dispersion depends on the internal structure of the medium.

The effect of ultrasonic cavitation on the thawing process generates the breaking up of ice crystals more quickly. At the same time, they melt at a faster rate in relation to the increase in intensity, power, and amplitude of the sound waves. The parameters that change with faster ice melting are the thawing rate, time, and start and endpoints of defrost. In general, the theory of thawing indicates that the rate of this process strongly influences water losses, changing its composition and altering the existing interactions between water and proteins. However, some studies [7, 15] have focused mainly on household defrosting and not commercial practices. Another factor to highlight is the long time required to thaw food conventionally, which can increase energy costs or require large amounts of water.

Authors such as Li et al. [16], Gan et al. [17], and Sun et al. [18] have explicitly worked with US applications for defrosting meat and seafood, varying application frequencies and comparing them with traditional methods. Regarding the analysis of thermal behavior during thawing, Hu et al. [9] and Kong et al. [19] present curves with several techniques, including infrared and microwave US, air, and immersion in water and only determine the total process time.

The reduction of structural damage due to the freezing of meat is directly dependent on the expansion in volume due to the change in physical state from water to ice. Reducing mechanical damage means keeping the ice crystals as small as possible, which can be achieved in two different ways: (1) with rapid freezing with cryogenic liquids or (2) the application of a cryoprotectant that forms an elastic polymeric network that traps water in small defined spaces and limits its growth intra and extracellularly.

Due to its high compatibility with meat and the cryoprotectors at low-temperature stability, waxy corn starch is a viable option for forming cryogels (C). This biopolymer has functional properties with various applications in the food industry, favoring the formation of structures with higher viscosity, thermostability, fluid texture, cohesiveness, low opacity, and thermoreversibility in postfreezing gels [20]. Previous studies [21, 22] have shown that the two-cycle waxy starch C can be considered a good cryoprotectant, due to the changes in its thermal behavior, related to enthalpies, temperatures, specific heat (Cp), and degree of structural order (close to 94.72%); as well as in the rheological behavior, regarding the formation of molecular interactions and the rearrangement in its structure, such as the formation of microporosities that are between 2.44 and 4.08 *μ*m. Therefore, applying C using high-intensity US, before conventional freezing in a chamber, reduces the changes in some quality attributes at the macro and microstructural level, generating a cryoprotective effect at the surface and internal level in the meat. This is achieved by coating the meat fibers, which allows for the reduction of structural damage, helping to reduce the size of the inter and intramyofibrillar spaces, thus avoiding alterations in the thermodynamics and chemistry of the proteins. These stabilizers induce the structural reorganization of the product, giving it greater solidity. However, in these studies, the method of thaw in the refrigeration chamber at 5°C ± 1.5°C for 6 h has remained constant, and no information has been found regarding the behavior of this cryoprotectant during defrosting or its conjunction with the application of US during the melting of ice crystals in the same process.

With the above, the present study focused on comparing a traditional method of defrosting pork cuts, such as water immersion versus US-assisted defrosting from a different and integral perspective using cryostructured material to improve the process conditions without causing significant effects on the final pork meat quality.

## 2. Materials and Methods

The study was divided into two parts. The first aimed at the selection of US conditions according to the thermal behavior at two levels (50 and 80% amplitude) and their effect on the shear force in thawed meat in a commercial refrigeration chamber for 12 h, and the second focused on the comparative analysis between raw and thawed meat, with and without application of waxy starch C and at the selected US level, evaluating its effect on some quality parameters.

### 2.1. Materials

Six-month-old castrated male pigs weighing approximately 100 ± 3 kg and 24 ± 1 h postmortem from a federal inspection type (FIT) certified farm in Cuautitlan Izcalli, State of Mexico, Mexico, were used. The animals were housed in a pen (4.9 × 2.0 m) with a concrete floor and a 0.5 m wide slatted manure area. Food and water were offered ad libitum. Samples of *Longissimus thoracis* muscle with a mean weight of 3.7 ± 0.3 kg were obtained from the 9th to 13th rib section. The chamber at 2°C was used for storage, and individual plates were cut of 3 × 3 × 2.5 cm with a weight of 21.79 ± 0.09 g, which was analyzed. The amount of sample subjected to freezing per spine was 1.8 ± 0.07 kg, equivalent to 82 samples, sufficient to determine the quality parameters in each part of the process. This was done on three loins of different pigs (three repetitions).

### 2.2. Preparation, Formation, and Diffusion of C

For the development of this research, the preparation, formation, and diffusion of C was carried out as indicated in the work of Coria-Hernández et al. [21], where maize waxy starch at 3% *w*/*w* (Firm-Tex, Ingredion, Mexico) was dispersed in water for 2 h at 40°C. It was frozen by immersion in liquid nitrogen up to −150°C ± 3°C for 10 min and thawed by immersion in water at 50°C ± 5°C for 30 min until two cycles were completed. The diffusion of the C inside the meat was carried out using high-intensity US with a UP200S (Hielscher, Teltow, Germany) at a power level of 20 W and constant frequency of 24 kHz, which was applied by immersion with a S14 probe sonotrode (max. submerged depth: 90 mm; tip diameter: 14 mm; max. amplitude: 80 *μ*m; acoustic power density: 105 W/cm^2^) at 0.8 cycles, 80% amplitude for 6 min at 25°C ± 2°C [22].

### 2.3. Freezing and Thawing of the Meat Samples

The freezing of the samples with C and without cryogel (WC) was performed by placing K-type thermocouples in the geometric center of each one of the three samples and subsequently freezing by indirect contact in liquid nitrogen, whose vapor reached up to temperature of −150°C ± 3°C, until the sample came to an inside temperature of −25°C. A thermal profile (temperature vs. time) was performed to obtain the initial freezing point (IFP) and the cooling rate. Samples were stored in a forced convection chamber in Torrey (Mexico) at −26°C ± 2°C for a maximum of 5 days.

Immersion thawing (It) was carried out in a WB05 thermostatic bath (PolyScience, Illinois, United States), with distilled water at 30°C ± 1°C, until the sample reached a final temperature of 25°C ± 1°C, performing the thermal profile with K-type thermocouples.

For US-assisted thawing, a Cole Parmer ultrasonic bath 8895-04 (Cole Palmer, Illinois, United States) was used at 37 kHz, 400 W, and with two levels of amplitude, which were analyzed to select the appropriate level according to the time of the thawing process, with less exudated juices and its effect on the required shear force, which is related to the structural impact of the meat by US treatment. A thermal profile was performed with the K-type thermocouples found in the frozen samples to obtain data on the interval of ice crystal melting temperature and thawing rate.

The initial melting point (IMP) was obtained on the first zone of the thermal profile during thawing. It was treated using the first derivative, allowing the inflection points to be determined. The second zone of the thermal profile determined the thaw rate. It was treated by employing a linear regression to obtain the slope representing the thawing rate.

### 2.4. Quality Parameters for Raw Material Control and Thawed Meat With C and WC and With and Without US

#### 2.4.1. pH of Meat

The pH was determined in raw and thawed meat with C and WC, and with and without US. Meat potentiometer HI99163 (Hanna Instruments, Rhode Island, United States) calibrated to three points with standard solutions at 25°C ± 1°C, according to Jansen [23], was used.

#### 2.4.2. Meat Color Profile

The color profile parameters were determined according to the methodology of Zonglin et al. [14] according to standardized conditions [24] with an EZ 4500 L spectrocolorimeter (HunterLab, Virginia, United States) with illuminant Type A, aperture of 8 mm and viewing angle of 10°, obtaining the values of the tristimulus coordinates in the Commission internationale de l'éclairage (CIE) system (*L*^∗^, *a*^∗^, and *b*^∗^). The recorded values determined the total color changes (Δ*E*).

#### 2.4.3. Percentage of Exudates

The method used was that of Cañeque and Sañudo [25], based on the European Commission [26], placing the samples between two absorbent papers measuring 5 × 5 cm and placing them in a press formed by two metal plates. A force of 2.5 kg_f_ was applied for 10 min, to remove the sample and record the weight of the absorbent paper. The exudate amount was obtained from the recorded data, using Equation (1).

(1) $$\begin{array}{c} \%\text{exudates}=\frac{\text{final weight of paper}-\text{initial weight of paper}}{\text{weight of sample}}\times 100 \end{array}$$

It was determined on all raw and thawed meat samples in their corresponding repetitions and replicates.

#### 2.4.4. Shear Force

The methodology described by American Meat Science Association (AMSA) [27] was followed using a CT3 texture analyzer (Brookfield, Massachusetts, United States). They placed the samples with the muscle fibers perpendicular to the 3 mm flat blade. The test speed was 1.50 mm/s with an activation load of 50 g_f_ at a controlled temperature. For the determination, the samples shall be left at room temperature to a surface temperature of 24°C ± 1°C.

#### 2.4.5. Surface Changes of Meat Samples

The surface appearance of meat samples with C and WC and with and without US was determined through photographs using an S2800 Coolpix high-resolution digital camera (Nikon, Tokyo, Japan) in a wooden lightbox (40 × 40 × 30 cm in dimension) equipped with two light sources at 6500 K and 45° inclination. The camera had a resolution of 20.1 megapixels and images were taken with a 2× zoom [28].

#### 2.4.6. Scanning Electron Microscopy (SEM)

The microstructure of each sample was observed at 330× using a JSM-6010LA SEM (Jeol, Tokyo, Japan), where the samples were cut perpendicular to the meat fibers, which were fixed with 2.5% glutaraldehyde for 30 min, and subsequently, they were dehydrated with ethanol (C_2_H_6_O, CAS number: 64-17-5) using a series of solutions of increasing ethanol concentration (50%, 70%, 90%, 95%, and 99%) and dried by freeze-drying under vacuum at 0.035 mBar in the collecting cabin, which was at −50°C for 24 h on a Freezone 4.5 (Labconco, Missouri, United States). Finally, the samples were ionized with a gold layer at 10 mA for 5 min in a Denton Desk V ion sprayer (Denton Vacuum, New Jersey, United States) [22].

### 2.5. Statistical Analysis

For all cases, measures of dispersion and central tendency were obtained followed by one-way or two-way ANOVA with their respective hypothesis testing (Tukey). Where necessary, linear and nonlinear regressions were performed (Minitab 16.0.1) (Penn State University, Pennsylvania, United States). A significance *p* value of < 0.05 was used to identify significant differences between treatments.

## 3. Results and Discussion

### 3.1. Selection of Ultrasonic Conditions (Amplitude 50% or 80%) in the Thawing With C

**Figure 1:**
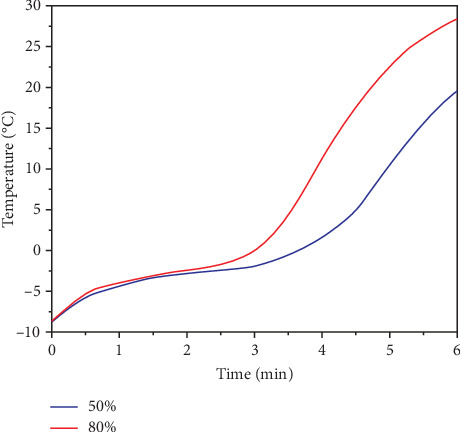
Thermal profile of thawing meat with cryogel in ultrasound at two amplitudes.

To select US treatment conditions in the first stage, the thermal profiles with amplitude of 50% and 80% were compared, as shown in Figure 1.

**Figure 2:**
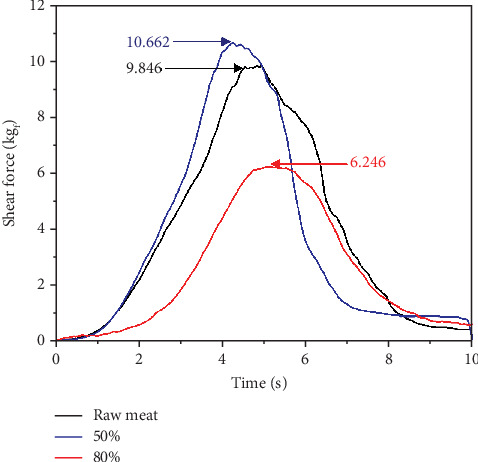
Shear force (kg_f_) on meat samples with cryogel and US. Raw meat and frozen meat with cryogel at 50% amplitude in US and frozen meat with cryogel at 80% amplitude in US.

At 6 min of US application with 80% amplitude, a final temperature of 28.2°C was reached with a melting rate of 0.83°C/min and a heating rate of 5.9°C/min; at 50%, a final temperature of 19.6°C with a melting rate of 0.65°C/min and a heating rate of 3.83°C/min, implying that there are better melting conditions at 80%. The mechanism of cavitation, with greater amplitude, produces more energy, leading to physical and chemical changes in biological structures that benefit the transfer of heat and the breaking of ice crystals [6]. However, although 80% showed advantages, a reduction of thawing time, it was observed that there was a more significant amount of exudates, indicating that there was a loss of 14.56% and 12.85%–50%, which is explained by the effect produced by the waves formed by US, which contribute to modifying protein structures, generating pronounced structural damage that favors a loss of exudates [29]. This is confirmed in the shear force graph shown in Figure 2, where the shear strength is less than 80% (6.246 kg_f_ and 10.662 kg_f_ at 50%), indicating more degree of structural damage originating from cavitation that induces fragmentation of the sarcomere structure [30].

With the results obtained, thawing with US at an amplitude of 50% was selected to continue the study.

### 3.2. Thermal Behavior During Freezing and Thawing of Meat

#### 3.2.1. Freezing

**Figure 3:**
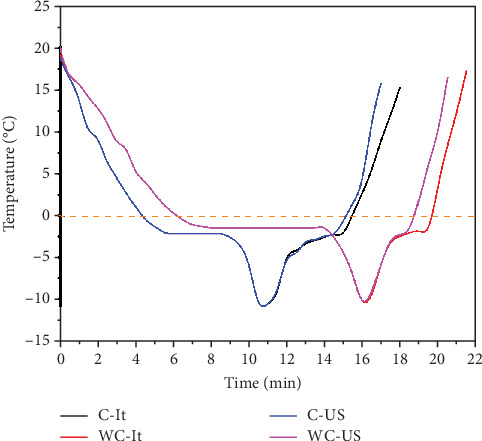
Thermal profile of freezing and thawing with cryogel in immersion and ultrasound. With cryogel with US (C-US), immersion cryogel (C-It), without cryogel with US (WC-US), and immersion without cryogel (WC-It).

The thermal behavior during freezing and thawing with the different treatments is shown in Figure 3, where the effect of C on the initial and final freezing and thawing temperatures is observed. The impact of the cryoprotector is remarked by decreasing the IFP, and the subcooling is extended by the interaction with water molecules through hydrogen bridges. These interactions reduce the degree of binding of water molecules and modify their three-dimensional structure, causing ice formation to be delayed, which would respect the principles of colligative properties.

The experimental IFP values are shown in Table 1. There is a decrease of 1 min in the freezing time and an increase in the cooling rate (2.81°C/min for raw meat and 4.31°C/min for meat added with C), which results from the interaction of the polysaccharide of the waxy starch C and water, where networks are formed that limit the freezing water, preventing osmotic outflows and increasing the surface area of contact by forming smaller ice crystals that promote more excellent heat transfer [31]. There is an increase in the time to reach the lowest subcooling temperature of 4 min when C was not added to the samples.

#### 3.2.2. Thawing: Crystal Melting and Rate of Pork Thawing With C by Immersion and US

The melting temperature is like a physical process where you go from a solid state to a liquid state, and this happens when you add energy in the form of heat, where the kinetic energy breaks down the intermolecular forces that held the crystal structure to a liquid state. As for thawing, this can also be seen in Figure 3, where the effect of C addition is evident.

Table 2 presents the results corresponding to the temperature range where the melting takes place, the time taken to melt the ice crystals considered only as a change in the latent heat of melting at an almost constant temperature, and the thawing rate where the transfer of sensitive heat occurs. These changes are explained by polysaccharide interaction in ice crystals, acting as a barrier that limits heat transfer, since without the addition of C, larger ice crystals are formed, where not only the convection heat transfer mechanism will predominate but also the conduction transfer between water molecules benefits [32].

The US increases the rate of thawing, which is explained by the phenomenon of cavitation that promotes heat transfer, where the impact waves generated provide a mechanism of forced convection, causing molecular collisions, which contribute to a rapid rise in temperature [6]. The thawing rate in the last zone is influenced by both the application of C and the use of US.

### 3.3. Quality Parameters in Defrosted Meat

#### 3.3.1. pH

pH, as a measure of the amount of free hydrogen ions (H^+^) in a solution and where the results obtained (Table 3) agree with that indicated by Coria-Hernández et al. [22]; Cheng, Song, and Kim [33]; and Leygonie, Britz, and Hoffman [34]. That shows that the quality of raw meat is acceptable and that the pH of frozen-thawed meat tends to be lower than before freezing (raw meat). The pH in tawed meat with C maintains a value close to that of raw meat because the polymer has ampholytic characteristics that do not modify the charges and maintain the balance of H^+^.

Analyzing the method of defrosting, in immersion, lower values are shown compared to US, and this is because the sample is kept at a high temperature for a longer time, which causes a protein denaturation, allowing the release of H^+^ and the consequent decrease of the pH. In addition, in this protein denaturation, there is a loss of water, which initiates an increase in the concentration of the solutes and a decrease in the pH [34]. The grouping column of Table 3 shows that the pH of samples added with C is statistically equal to the pH of raw meat; however, there are significant differences concerning samples that did not contain C. Comparing the results of the defrosting methods does not show statistically significant differences, indicating that the main factor influencing pH is the addition of C.

#### 3.3.2. Color Profile Parameters

The means of each coordinate in the CIE system (*L*^∗^, *a*^∗^, and *b*^∗^) and the total color difference (Δ*E*) were obtained. Table 3 shows only the comparison of the last, which coincide with what was reported by Coria-Hernández et al. [22] and Cheng, Song, and Kim [33]. Color profile parameters are related to water holding capacity (WHC), since myoglobin is a water-soluble protein that is lost along with the fluids exuding from meat and modifying the values of *a*^∗^ and *b*^∗^. Differences in Δ*E* are mainly related to the quantity and *redox* state of the *heme* group of myoglobin, where the oxidation rate of the pigment increases since increasing the temperature accelerates the oxidation–reduction reactions of the muscle pigments, thus contributing to the more significant discoloration of the meat; therefore, the color will be less stable than in raw meat. However, it has been shown that the most relevant changes in meat occur in luminosity [35].

In the latter, there is no significant difference (*p* < 0.05) between the application of C and US and raw meat, contrary to the other treatments. This is explained by the fact that as the percent of exudates decreases, there is an increase in the intrinsic birefringence; that is, the anisotropic properties of the molecules modify the elasticity of the light waves, which is represented by a dispersion of the light and an increase in the luminosity (*L*^∗^). Spaces generated between myofibrils by protein denaturation, WHC reduction, and sarcolemma disruption are critical structural changes that increase reflected light [36]. However, the thresholds Δ*E* depend on the characteristics of the sample, and there are no accurate data on the color difference for meat products, so the differences could be considered subjective [35].

#### 3.3.3. Percentage of Exudates and WHC

WHC is directly related to the quantity of exudates and represents the ability of meat to retain water even when external pressures are applied. It is also associated with pH, which can be seen in the results of Table 3, where the values represent water loss expressed as a percentage and follow a similar trend to pH. There is a more significant loss in samples without the addition of C and in It. However, statistically, it is different from that of raw meat. This is explained by the fact that pH is one of the factors involved in modifying the isoelectric point in proteins, thus promoting the protein denaturation and affecting their structure. In addition, this change in pH, combined with the increase in temperature, causes enzyme reactivation, mainly of cathepsin, which causes protein degradation, leading to more significant fluid outflow [36]. Likewise, the US facilitates heat transfer without causing considerable damage to protein structures. According to Stadnik and Dolatowski [37] and Jayasooriya et al. [38], the reduction of this parameter is due to the activation of calpain enzymes, responsible for the maturation of meat and, therefore, the loss of water localized in the intramiofibrillar spaces of the muscle, suggesting that sound waves contribute to the modification of protein structures. Concerning the immersion method, where the thawing rate is reduced, Dang et al. [39] have shown that the rate at which the temperature decreases influences the amount of water lost during thawing because they produce a more significant denaturation of proteins and causes a greater rupture of muscle fibers.

#### 3.3.4. Shear Force

**Figure 4:**
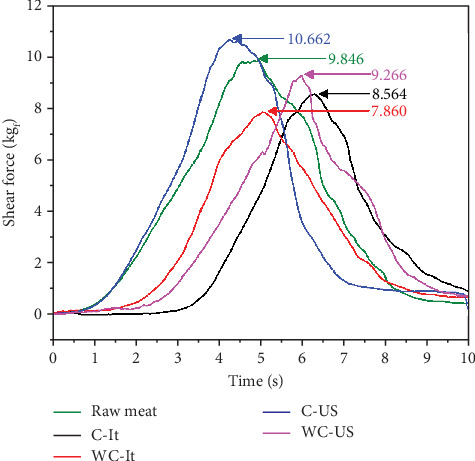
Shear force profiles of meat samples treated at different conditions.

Similarly, the shear force was evaluated, obtaining that the maximum shear point of raw meat is 9.846 kg_f_, which agrees with the data reported in the literature by Rees, Trout, and Warner [40]. Chacón [41] mentions that the hardness of meat is mainly due to muscle proteins: connective tissue (collagen, elastin, and reticulin), myofibrillar (actin and myosin), and sarcoplasmic, as well as fat content and % of exudates.  Figure 4 shows a graph representing each treatment, indicating the cutting resistance, representing the maximum point. Table 3 shows the mean cutting force values of the meat samples.

It is observed that the treatments with added polymer present superior resistance to cutting, which proves that the C fulfills its function as a protector, providing more stability at the structural level. The phenomenon of cavitation produced by US, which does not destroy the meat matrix, unlike defrosting by immersion, is demonstrated. It is verified that the conditions of meat with C and WC thawed in US do not show significant differences compared to raw meat. It is indicated that the use of C does not represent a statistically different (*p* > 0.05) effect between samples, compared to the thawing method, where there are large significant differences. The above shows that thawing is the main factor influencing the shear force.

Likewise, changes due to US treatment induce fragmentation of the sarcomere structure, increasing the softness of the meat. On the other hand, Stadnik and Dolatowski [37] have shown that US does not cause softening in meat samples; this may be due to the use of low-frequency US waves, which may be insufficient to induce changes in meat texture.

#### 3.3.5. Surface Changes of Meat Samples

**Figure 5:**
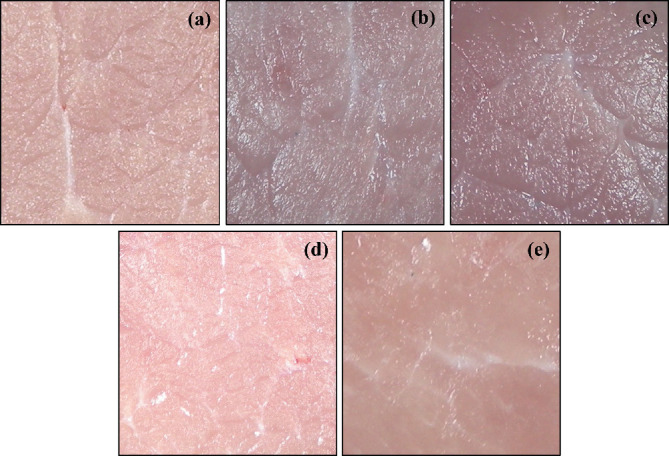
Surface of meat samples treated under different conditions. (a) Raw meat, (b) C-It, (c) WC-It, (d) C-US, and (e) WC-US.

An important characteristic to consider when thawing meat is its surface appearance and the changes that are caused by the different treatments to which it was subjected. This is in order to determine, to a certain degree, the changes in the proteins and their relationship with the quality attributes evaluated, such as color, WHC, and shear force.  Figure 5 shows the images of the raw meat and of the different thawing treatments (immersion and US), where it is clearly observed that there are variations in the surface color.

Zhang et al. [4] and Qiu et al. [6] report similar results regarding the effects of It and ultrasonic cavitation. In this sense, the data obtained agree with the Δ*E* values, since it is observed that the samples that were immersed in water (Figures 5(b) and 5(c)) present the greatest total color changes (of the order of 9–11 units) at difference from the samples that were subjected to US thawing (Figures 5(d) and 5(e)), where the pigments behaved more stably, as reported by Xu et al. [13] and Coria-Hernández et al. [42]. Likewise, the shear force values are directly related to the surface changes, specifically in this case; the samples treated with US are those that have better appearance and greater shear resistance. Regarding the addition or not of C, it was found that it did not develop a determining effect on the appearance of the samples; however, it is important to highlight that Figure 5(d) presents very similar characteristics with respect to raw meat, so it is considered that the synergy between the thawing method and the addition of the cryoprotectant was what kept the surface of the meat in very similar conditions.

#### 3.3.6. SEM Studies

**Figure 6:**
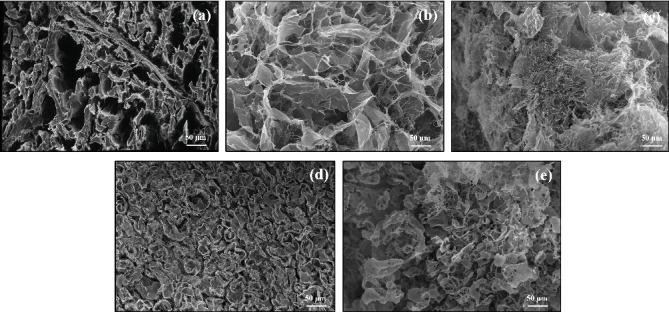
Microstructure of meat samples treated under different conditions. (a) Raw meat, (b) C-It, (c) WC-It, (d) C-US, and (e) WC-US.

During recent years, the use of SEM in the field of food technology has served as support to study the effects of various technological processes, such as freezing, thawing, and the application of US, which allows detailing important questions about the microstructure and surface and internal morphology of foods. In this sense, the micrographs of the meat samples (Figure 6) allow us to more clearly visualize the damage that could have occurred due to the addition or not of the C and the thawing process (US or immersion).

That is why, in accordance with the results obtained from the images collected at the surface level, it is clearly observed that thawing by immersion (Figures 6(b) and 6(c)) has a negative effect on the meat fibers, causing the internal structure to deteriorate and collapse, even regardless of whether there is a protective coating generated by the addition of C. This is primarily due to the fact that It favors modifications in the proteins responsible for maintaining the structure of meat fibers in adequate conditions, compared to samples that were subjected to US-assisted thawing (Figures 6(d) and 6(e)), where it can be seen that the combination with C produces an important synergy, which allowed the microstructure to be maintained in conditions very similar to those of raw meat without treatment and with less damage.

Authors such as Zhu et al. [1], Li et al. [5], Qiu et al. [6], Zhang et al. [43], and Wu et al. [44] describe that the use of sonoprocessing as an assistive technology in the defrosting of meat has less impact on myofibrillar and connective tissue proteins, which translates into fewer changes at the internal and superficial levels, minimally affecting its functionality, which has a significant impact on aspects of final quality, which is why they recommend that under adequate conditions of power, amplitude, and cycles, the microstructure of the meat will remain practically intact.

## 4. Conclusions

Based on the conditions under which the experimental tests were carried out and based on the results obtained in terms of heat transfer and quality parameters of this investigation, the following conclusions can be drawn.

Adding C to pork and US-assisted thawing showed a favorable effect, decreasing the time, increasing the thawing rate, and influencing some quality parameters.

The quality attributes evaluated in C and thawed meat samples in US indicate no perceptible changes for consumers.

Finally, it can be verified that, in pork with waxy starch C, the frequency and amplitude of US during thawing promote thermal transfer, which is reflected in the decrease of thawing time, in addition to improving WHC and decreasing changes in pH, surface color and shear force resistance, resulting in less damage to muscle structure compared to conventional It. In this sense, we found that combining C and US in the freezing-thawing process increases the efficiency of the processes, minimizing changes in some quality attributes. These results allow us to establish that we could work on the design of a unit that, like a microwave oven, could carry out the functions of a household defroster.

## Figures and Tables

**Table 1:** IFP values in raw meat and cryogel-US compared to references [22].

	**Raw meat**		**Meat with cryogel-US**	
	**Obtained**	**Reference value**	**Obtained**	**Reference value**
IFP (°C)	−1.40 ± 0.01	−1.26 ± 0.00	−2.10 ± 0.00	−2.08 ± 0.01

*Note:*Mean ± standard deviation.

**Table 2:** IMP values and thawing rate of the different treatments.

**Treatment**		**Ice melting temperature (°C)**	**Ice melting time (min)**	**Thawing rate (°C/min)**
C	US	−5.1 ± 0.0 to −2.2 ± 0.0^a^	2.5 ± 0.2^a^	4.94 ± 0.03^a^
	It	−3.3 ± 0.2 to −2.0 ± 0.1^b^	3.0 ± 0.1^a^	3.05 ± 0.18^b^
WC	US	−0.2 ± 0.0 to −1.4 ± 0.1^c^	1.0 ± 0.0^c^	4.26 ± 0.02^a^
	It	−3.2 ± 0.3 to −1.2 ± 0.5^b^	2.0 ± 0.1^b^	2.38 ± 0.02^c^

*Note: *Mean ± standard deviation. Means with different letters in the same column are statistically different (*p* < 0.05).

Abbreviations: C, cryogel; It, immersion thawing; US, ultrasound-assisted thawing; WC, without cryogel.

**Table 3:** Results of the variables determined in the samples subjected to different treatments.

**Sample**	**pH**	**WHC (%)**	Δ**E**	**Shear force (kg**_**f**_ **)**
Raw meat	5.47 ± 0.04^a^	89.91 ± 0.57^a^	—	9.846 ± 0.340^b^
C-US	5.44 ± 0.01^a^	87.17 ± 1.03^b^	5.38 ± 0.74^b^	10.662 ± 0.191^a^
C-It	5.41 ± 0.02^a^	82.15 ± 1.35^b^	9.74 ± 1.02^a^	8.564 ± 0.360^b^
WC-US	5.34 ± 0.02^b^	83.69 ± 1.09^c^	8.70 ± 1.33^a^	9.266 ± 0.052^c^
WC-It	5.31 ± 0.04^b^	79.94 ± 0.66^d^	10.88 ± 1.87^a^	7.860 ± 0.371^c^

*Note:* Means with different letters in the same column are statistically different (*p* < 0.05).

Abbreviations: C, cryogel; It, immersion thawing; US, ultrasound-assisted thawing; WC, without cryogel.

## Data Availability

The data used to support this study are provided within the article.
